# *Emergomyces*: The global rise of new dimorphic fungal pathogens

**DOI:** 10.1371/journal.ppat.1007977

**Published:** 2019-09-19

**Authors:** Ilan S. Schwartz, Nelesh P. Govender, Lynne Sigler, Yanping Jiang, Tsidiso G. Maphanga, Barbra Toplis, Alfred Botha, Karolina Dukik, J. Claire Hoving, Jose F. Muñoz, Sybren de Hoog, Christina A. Cuomo, Robert Colebunders, Chris Kenyon

**Affiliations:** 1 Division of Infectious Diseases, Department of Medicine, Faculty of Medicine and Dentistry, University of Alberta, Edmonton, Alberta, Canada; 2 National Institute for Communicable Diseases, a Division of the National Health Laboratory Service, Johannesburg, Gauteng, South Africa; 3 Department of Clinical Microbiology and Infectious Diseases, University of the Witwatersrand, Johannesburg, Gauteng, South Africa; 4 Department of Biological Sciences, Faculty of Sciences, University of Alberta, Edmonton, Alberta, Canada; 5 Westerdijk Fungal Biodiversity Institute, Utrecht, the Netherlands; 6 Department of Dermatology, The Affiliated Hospital, Guizhou Medical University, Guiyang, China; 7 University of the Free State, Bloemfontein, Free State, South Africa; 8 Department of Microbiology, Stellenbosch University, Stellenbosch, Western Cape, South Africa; 9 Institute of Infectious Diseases and Molecular Medicine, University of Cape Town, Cape Town, Western Cape, South Africa; 10 Broad Institute of MIT and Harvard, Cambridge, Massachusetts, United States of America; 11 Center of Expertise in Mycology of RadboudUMC/ Canisius Wilhelmina Hospital, Nijmegen, The Netherlands; 12 Global Health Institute, University of Antwerp, Antwerp, Belgium; 13 Clinical Sciences Unit, Institute of Tropical Medicine, Antwerp, Belgium; 14 Department of Medicine, University of Cape Town, Cape Town, Western Cape, South Africa; Geisel School of Medicine at Dartmouth, UNITED STATES

Unknown until recently, the genus *Emergomyces* is composed of dimorphic fungi with human pathogenic potential. Five described species have been implicated as causes of disease in immunocompromised persons on at least 4 continents. In some regions, such as southern Africa, this disease, emergomycosis, is now the most commonly diagnosed dimorphic fungal infection. Herein, we review the history of these fungi, clinical features and pathogenesis of disease, what is known of their biology, and future research directions.

## Origin story and current status

In 1994, an Italian woman with advanced HIV disease and widespread cutaneous lesions was diagnosed with an atypical disseminated mycosis. A biopsy of skin tissue demonstrated small budding yeasts, resembling those of *Histoplasma capsulatum*, in addition to larger pleomorphic cells. Moreover, the fungus that grew in culture had a dissimilar microscopic appearance [1, 2]. The conidia were arranged in complex “florets” on slightly swollen stalks reminiscent of *Emmonsia crescens* or *Ea*. *parva*, fungi for which the thermodependent phase is characterized by swollen, thick-walled, nonreplicating cells called adiaspores, usually observed in the lungs of small terrestrial mammals in a disease called adiaspiromycosis [3]. Genetic analysis suggested the clinical isolate was related to *Ea*. *crescens*, and the fungus was therefore described in 1998 as *Ea*. *pasteuriana* [2].

In 2013, following the introduction of molecular identification procedures in some South African laboratories, a dimorphic fungus most closely related to *Ea*. *pasteuriana* was found to be the cause of a disseminated mycosis in patients with advanced HIV disease [4]. Since then, infection with this fungus has been established to comprise the most frequently diagnosed dimorphic mycosis in South Africa [5–7]. In light of these reports, global collections were re-examined for dimorphic *Emmonsia*-like fungi. Archived isolates were examined phenotypically and with genetic analyses based on ribosomal DNA sequences, ultimately leading to a taxonomic revision within the family Ajellomycetaceae [8, 9]. In brief, *Ea*. *parva*, the type species of *Emmonsia*, was transferred to the genus *Blastomyces* (as *B*. *parvus*), and the genus *Emmonsia* was more narrowly defined to include *Ea*. *crescens* and *Ea*. *soli*, the latter currently known only from soil [8, 9]. A new genus, *Emergomyces*, was created to accommodate *Emmonsia*-like systemic dimorphic pathogens related to *Es*. *pasteurianus* (formerly *Ea*. *pasteuriana*) and characterized in the thermodependent phase by small yeast cells with narrow-based buds [8, 9].

**Fig 1:**
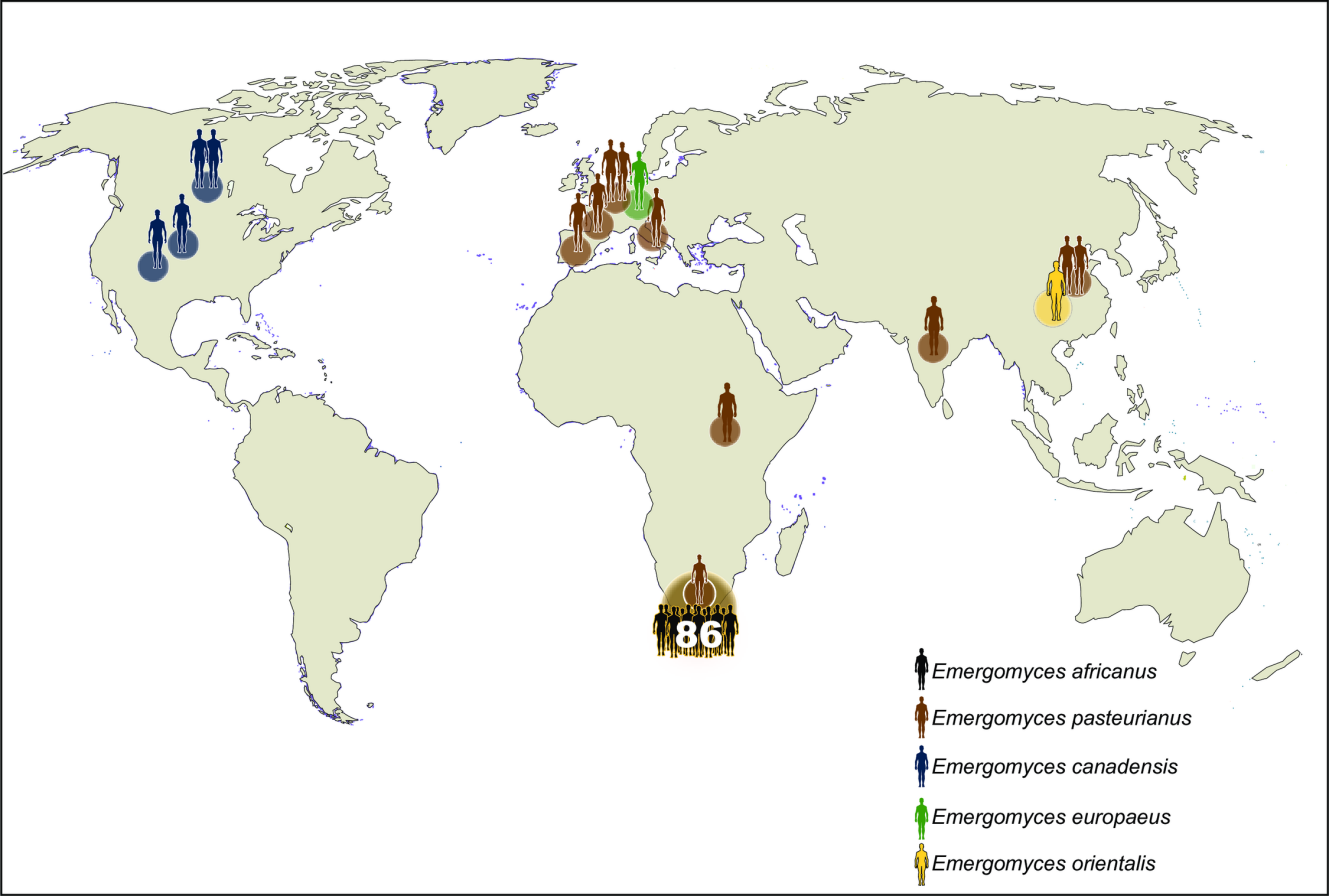
Global geographic distribution of reported cases of emergomycosis [1, 4–7, 10–19, 28–30]. Each icon represents a single case except for *Emergomyces africanus* in South Africa, as indicated. Map created by Institute of Tropical Medicine, Antwerp.

Five species are now described within *Emergomyces* [8, 9], and cases of disease have been reported globally (Fig 1). *Es*. *pasteurianus*, the type species, has been reported from Europe (including from Italy [1], Spain [10], France [ex-Georgia] [11], and the Netherlands [12]), Asia (China [13, 14] and India [ex-Nepal] [15]) and Africa (Uganda [ex-Rwanda] [16] and South Africa [9]). *Es*. *africanus* has been reported from South Africa and Lesotho [4, 9]. *Es*. *canadensis* has been reported from Canada (Saskatchewan) and the United States (Colorado and New Mexico) [17]. *Es*. *orientalis* has been reported from China [18], and *Es*. *europaeus* has been reported once from Germany [19].

The earliest known isolate among these *Emergomyces* species was from 1992 [17]. It remains unclear if these fungi have truly emerged or whether they are only now recognized because of an increase in the number of susceptible hosts, improved microbiology capacity, and/or the widespread adoption of molecular identification techniques in clinical and research laboratories.

## Ecology

**Fig 2:**
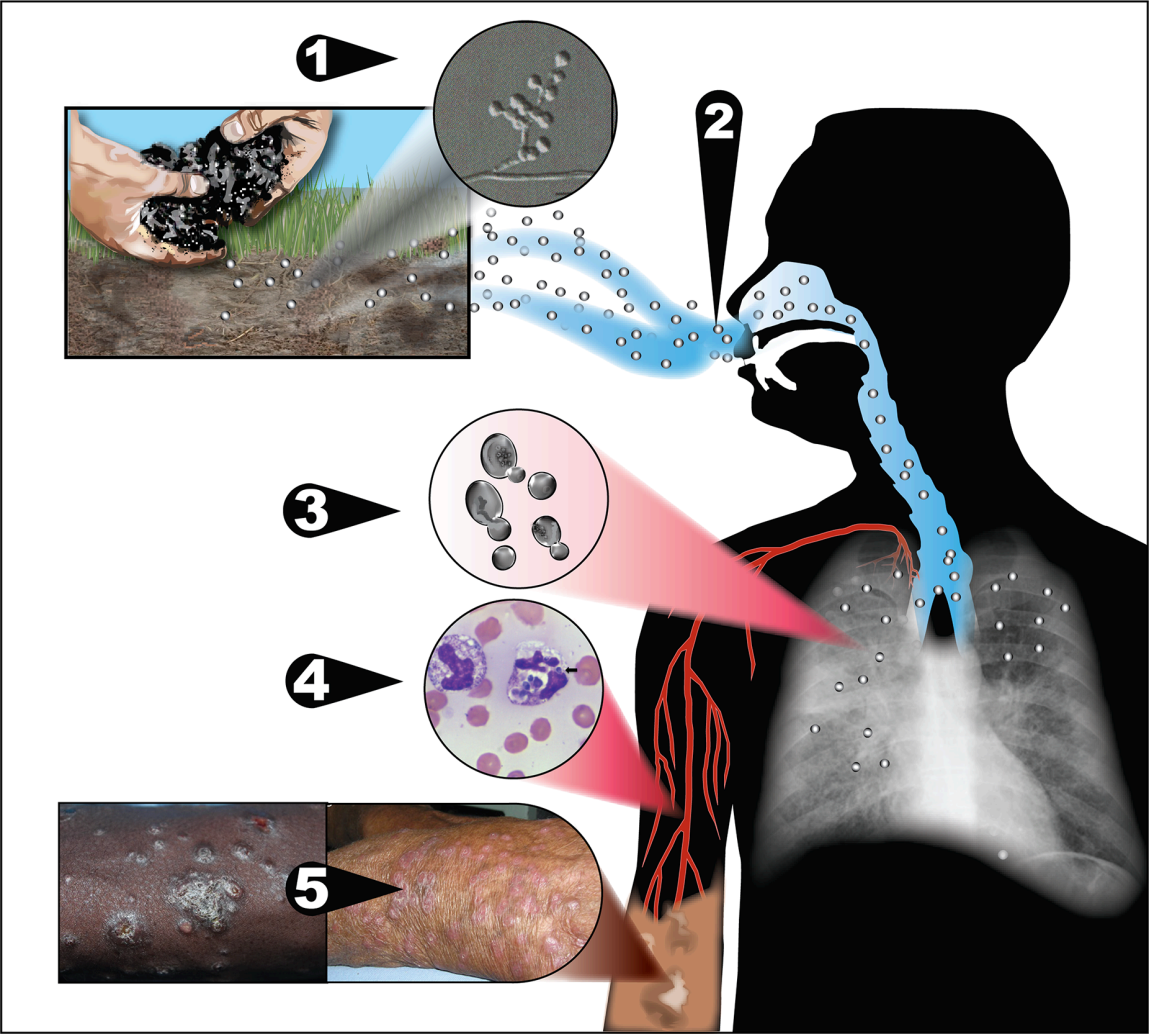
Potential model for exposure and pathogenesis of *Emergomyces*. 1) *Emergomyces* species exist in soil in a mold phase, from where 2) conidia are released and aerosolized. 3) Upon inhalation by humans, the conidia undergo a temperature-dependent transformation in the lungs to yeast-like cells that replicate by budding and are capable of causing pulmonary disease in susceptible individuals. 4) Yeast-like cells disseminate hematogenously in macrophages throughout the body, causing extrapulmonary disease. 5) Cutaneous disease is most frequently reported, although virtually any body site can be affected.

As with other systemic dimorphic fungi, infection is presumed to occur from inhalation of conidia, followed by a temperature-dependent transformation to a yeast-like phase that is capable of causing disease and of extrapulmonary dissemination (Fig 2).

Studies in search of an environmental reservoir for *Emergomyces* species have focused on *Es*. *africanus* in South Africa due to the large number of cases there [20–22]. *Es*. *africanus* DNA was detected in 30% of soil samples tested in the Western Cape Province, including from a wide range of soil habitats, such as soils associated with human activities like agriculture and horticulture and soils associated with plants endemic to the Cape Floral Kingdom. However, to date, the fungus has not been successfully isolated in culture from the environment, even with passage of soils through mice [20]. *Es*. *africanus* DNA was also detected in 10% of air samples collected in an urban location in the Cape Town metropole [21]. Naturally occurring infections of animals have not been demonstrated [22].

## Emergomycosis

Disease caused by *Emergomyces* infection has been called emergomycosis (formerly disseminated emmonsiosis), and similarities have been noted among cases caused by different species. In general, patients with reported emergomycosis have been immunocompromised, including with HIV infection, solid organ transplantation, hematological malignancies, and immunosuppressant use [23]. Most patients with *Es*. *africanus* infection have had cutaneous lesions, which most commonly appear as papules, plaques, nodules, or ulcers, typically with widespread distributions [4, 6]. Pulmonary disease is also common: 86% of patients had abnormal chest X-rays in a series from South Africa [6]. Chest radiograph abnormalities have included diffuse reticulonodular disease, consolidation, effusions, and/or lymphadenopathy [6]. Other sites of disease that have been reported include the gastrointestinal tract, liver, lymph nodes, and bone marrow [6]. Limited pulmonary disease has rarely been described, observed in the sole reported case of disease caused by *Es*. *europaeus* [19]. For other species, all reported cases have involved disseminated disease. This may reflect a diagnostic bias in clinical practice due to late disease presentation and limited access to more invasive (e.g., bronchoscopic) pulmonary sampling in resource limited settings or reporting bias in the medical literature.

The diagnosis of emergomycosis can be made by biopsy of affected tissue for histopathology and fungal culture. Histopathology findings include small (2–5-μm) yeasts with narrow-based budding, best seen with fungal stains [4]. The findings are insufficiently distinct from *H*. *capsulatum* to allow definitive identification from histopathological appearance alone [5]. The diagnosis can be confirmed by culture of *Emergomyces* species from clinical samples. Where fungal cultures are negative (or omitted), PCR of fresh, affected tissue using amplification and sequencing of the internal transcribed spacer (ITS) can establish the correct diagnosis [6, 9].

*Emergomyces* species grow readily on standard fungal media (e.g., Sabouraud agar, malt extract agar, or potato dextrose agar), incubated at 24–30°C. Colonies are yellowish white to tan, initially glabrous, becoming powdery, slightly raised, and furrowed, and reach diameters of 2.5 to 3.5 cm in 3 weeks. Microscopically, *Emergomyces* spp. are characterized in the mold phase by slender conidiophores that arise from hyphae at right angles and form “florets” of short secondary conidiophores bearing single small subspherical conidia (see Fig 2, step 1 [inset]). Conversion from the mold to the yeast phase occurs readily when colonies are streaked onto potato dextrose agar or malt extract agar and incubated at 35°C [8, 9, 17].

Clinical microbiologists should be aware that *Emergomyces* species can cross react with a commercial DNA probe for *B*. *dermatitidis* [17]. There are neither sensitive nor specific serological tests nor biomarkers for the diagnosis of emergomycosis, though cross-reactivity can be observed with tests for related fungal infections [5, 6]. *Emergomyces* species may cross react with *Histoplasma* urinary antigen tests [5, 6], but a negative test cannot exclude the diagnosis: in a series of 10 patients with culture-proven emergomycosis, only 3 tested positive by *Histoplasma* urinary antigen test [5].

Current treatment recommendations for emergomycosis are based on observational studies and expert opinion and are the same as those for patients with histoplasmosis [6, 23]. Initially, immunocompromised patients with emergomycosis should be treated with amphotericin B for 10–14 days; where available, liposomal formulations are preferred over deoxycholate because of a more favorable toxicity profile. Thereafter, patients should be treated with itraconazole or another newer azole for 12 months pending immune reconstitution. Fluconazole should be avoided because high minimum inhibitory concentrations have been observed [7, 17, 24]. Among HIV-infected persons with emergomycosis who are antiretroviral therapy (ART)–naive (or on a failing ART regimen), the optimal timing of ART initiation (or modification) has not been established.

## Pathogenesis

There remains much to learn about the virulence factors of these fungi and pathogenesis involved in infection. Known virulence factor genes of dimorphic fungi are conserved in *Es*. *africanus* and *Es*. *pasteurianus* [25]. The role of these in *Emergomyces* pathogenesis still needs to be evaluated. Strains of *Es*. *africanus*, *Es*. *europaeus*, and *Es*. *pasteurianus* (but not *Es*. *canadensis* or *Es*. *orientalis*) express urease [8, 26], a known virulence factor for some pathogenic fungi like *Cryptococcus neoformans* and *C*. *gattii*. Experimental infections have demonstrated susceptibility of golden hamsters and mice [20, 27]. Schwartz and colleagues found that intraperitoneal inoculations with *Es*. *africanus* were fatal to wild-type mice at doses of 10^6^ conidia, whereas lower doses did not cause disease (although the organism could still be cultured from their livers and spleens with inoculae as low as 10^2^ conidia) [20]. Moreover, C57BL/6 mice were more susceptible to disease than BALB/c mice [20]. Further work is underway to understand the pathogenesis of disease and the immunology of infection.

## Future directions

There are many unresolved questions about *Emergomyces*. The true geographic range of *Emergomyces* species remains speculative, given the sporadic reports of disease from areas with limited mycological diagnostic capacity. For example, a case of emergomycosis caused by *Es*. *pasteurianus* in Uganda was diagnosed after a visiting doctor returned to Germany with a skin biopsy specimen that was collected from a Ugandan patient with a disseminated mycosis [16]. There, the diagnosis was made by nucleic acid amplification and sequencing, technology lacking in most African settings. It is presumed that this case and those diagnosed in South Africa represent the “ears of the hippo” on the African continent and that many other cases there and possibly on other continents go unrecognized. The development of an affordable, accessible, and feasible diagnostic test for emergomycosis should be prioritized to enable the diagnosis in places where the disease is widespread and to detect the presence of it elsewhere for epidemiological surveillance.
